# Assessing air quality index awareness and use in Mexico City

**DOI:** 10.1186/s12889-018-5418-5

**Published:** 2018-04-23

**Authors:** Timothy C. Borbet, Laura A. Gladson, Kevin R. Cromar

**Affiliations:** 1 Sackler Institute, New York University School of Medicine, 423 East 23rd Street, 6027 West, New York, NY 10010 USA; 2 Marron Institute of Urban Management, New York University, 60 5th Avenue, 2nd Floor, New York, NY 10011 USA

**Keywords:** Air quality index, Risk communication, Behavior modification, Air pollution

## Abstract

**Background:**

The Mexico City Metropolitan Area has an expansive urban population and a long history of air quality management challenges. Poor air quality has been associated with adverse pulmonary and cardiac health effects, particularly among susceptible populations with underlying disease. In addition to reducing pollution concentrations, risk communication efforts that inform behavior modification have the potential to reduce public health burdens associated with air pollution.

**Methods:**

This study investigates the utilization of Mexico’s IMECA risk communication index to inform air pollution avoidance behavior among the general population living in the Mexico City Metropolitan Area. Individuals were selected via probability sampling and surveyed by phone about their air quality index knowledge, pollution concerns, and individual behaviors.

**Results:**

The results indicated reasonably high awareness of the air quality index (53% of respondents), with greater awareness in urban areas, among older and more educated individuals, and for those who received air quality information from a healthcare provider. Additionally, behavior modification was less influenced by index reports as it was by personal perceptions of air quality, and there was no difference in behavior modification among susceptible and non-susceptible groups.

**Conclusions:**

Taken together, these results suggest there are opportunities to improve the public health impact of risk communication through an increased focus on susceptible populations and greater encouragement of public action in response to local air quality indices.

**Electronic supplementary material:**

The online version of this article (10.1186/s12889-018-5418-5) contains supplementary material, which is available to authorized users.

## Background

Air quality has emerged as a global public health concern due to decades of research providing evidence of its wide-reaching human health effects. Air pollution is considered the number one environmental cause of premature mortality and current estimates attribute over 1 million deaths per year to exposure [1]. While basic science and epidemiological studies have linked components of air pollution (such as particulate matter and ozone) with detrimental health effects [2–4], there is still a gap in the existing knowledge regarding how to intervene and limit human exposure in highly polluted areas [5, 6].

The Mexico City Metropolitan Area (MCMA), an expansive urban region with a population of over 20 million, has a long history of air pollution which has impacted the acute and chronic health of those living in its borders [7–9]. Mexico City has some of the worst air quality in the Western Hemisphere, a result of its unique geography and significant urban expansion in response to an ever-growing population [10, 11]. While air quality in this region has improved markedly since new policies were passed in the early 1990s, pollutant reductions have since reached a standstill and fine particulate matter pollution has actually worsened in recent years [12].

**Fig. 1:**
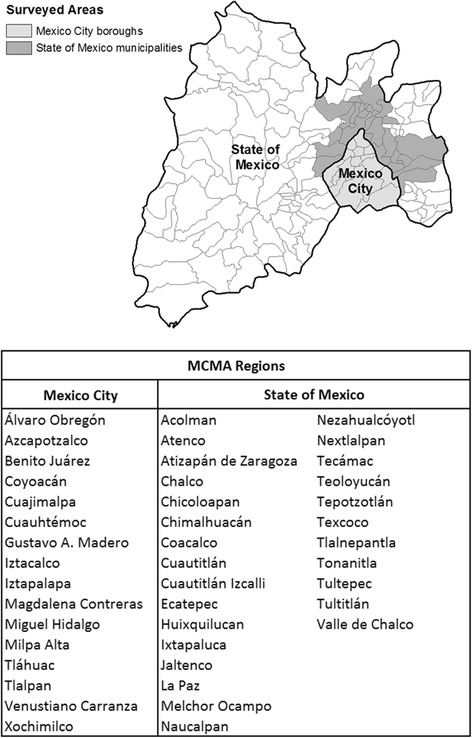
Mexico City Metropolitan Area. Survey participants were from Mexico City boroughs or State of Mexico municipalities, as shown in the table. These regions are labeled and outlined in black in the map, and surveyed areas are shown for the State of Mexico (dark grey) and Mexico City (light grey). This map was generated by the authors using ArcGIS software [30]

The MCMA is composed of the boroughs and municipalities of Mexico City, containing nearly half of the area’s population, as well as some located within the State of Mexico (see Fig. 1). Within the MCMA, Sistema de Monitoreo Atmosferico de la Ciudad de México monitors ambient air concentrations of six criteria air pollutants that have adverse human health and environmental effects: ground level ozone (O_3_), carbon monoxide (CO), sulfur oxides (SO_x_), nitric oxides (NO_x_), lead, and particulate matter [13, 14]. The Índice Metropolitano de la Calidad del Aire (IMECA) reports daily air quality based on these six pollutants and assigns a score between 0 and 500 for each air quality report [15]. Scores in the range of 101–150 reflect atmospheres that are unhealthy for sensitive populations, such as young children and older adults with underlying cardiac or pulmonary disease; scores of 151–200 indicate air considered harmful to the entire population; IMECA values > 200 indicate a state of emergency, wherein the entire population is at risk for adverse health effects. The highest measured value for an individual pollutant will determine the IMECA value for that particular day. This information is updated every hour and forecasted for future days to be made available to the public via media outlets including the internet, social media, web-based applications, and news outlets [16].

A strategy to reduce health effects associated with poor air quality has been to enhance public awareness and education of air quality and monitoring tools. Three key components of air pollution awareness include knowledge of air quality reports, what these reports mean, and how to use them to reduce exposure [17]. Air quality reports provide guidelines to reduce outdoor activities among susceptible groups during severe pollution events. However, if this information does not reach its target audience, then the method of communication is ineffective. While a handful of studies in the U.S. have considered public awareness of air quality indices, how these impact behavior, and how individuals respond to air pollution [5, 18–20], more extensive global research is needed. Deficits exist in the understanding of how people learn about air quality monitoring data, and the effectiveness of risk communication with respect to air pollution. This is pertinent to the multiple countries across the Americas, Europe, Asia, and Australia that utilize air quality index risk communication systems to inform the public about poor air quality [21].

To determine public use of air quality reports, the 2005 Behavioral Risk Factor Surveillance Studies (BRFSS) conducted in the United States (Kansas, Colorado, Indiana, Massachusetts, and Wisconsin) assessed knowledge of the U.S. Air Quality Index (AQI) and factors associated with this awareness. The survey reported that 43–53% of all respondents had heard of the AQI [5, 22]. The results supported the belief that people with preexisting respiratory diseases in their families were more likely to know about the AQI and to modify their behavior accordingly. Similarly, Potter and Perveen [5] found healthcare providers to be an important resource in helping patients to modify their behavior in response to poor air quality.

Risk communication systems produced by health agencies provide the public access to information with the aim to minimize exposure to harmful environmental factors via behavior adjustments when the health benefits outweigh the opportunity cost [23]. Presently, there is a limited understanding of public awareness of air quality indices and how these reports impact behavior, particularly outside the U.S. To address this deficiency, this study used data obtained through a phone-based survey to assess the MCMA general population’s knowledge of IMECA, what factors contributed to IMECA awareness, and whether this knowledge of the air quality index led to a behavior response. It was expected that study results would gauge IMECA awareness and use, and help inform future policy measures to be more successfully applied. Although this study utilized data collected within the MCMA, the results have relevance to other areas of the world that use air quality reports as a method of intervention to limit human exposure to air pollution. These data also enhance the field’s knowledge of air quality index effectiveness in a large metropolitan area outside the United States and analyze index awareness in the context of respiratory illness in the home, providing much needed insight into the use and impact of alert systems in targeting vulnerable populations.

## Methods

### Survey data

The polling company Parametría (Mexico City, Mexico) administered a voluntary phone-based survey between June 29 and July 2, 2015. A total of 803 individuals participated in the survey, with 427 participants from boroughs within Mexico City and 376 participants residing in municipalities from the State of Mexico. Both areas are part of the MCMA (see Fig. 1). Individuals were selected for the survey using a random sampling matrix to ensure even geographical coverage of respondents. A power analysis was performed to ensure adequate sampling to confidently determine a difference of 6% with a type I error rate of 1% and power of 0.8. Responses to a set of predetermined questions, age, gender, and occupation were recorded from consenting adults living in the MCMA. Inclusion was limited to adults to respond on behalf of the household, and individuals under 18 were not surveyed. The data was provided to researchers de-identified to leave no personal information or means for follow-up contact with respondents.

### Survey questions

Survey questions included modified and Spanish-translated versions of the United States 2005 Behavior Risk Factor Surveillance Systems (BRFSS) found in the Kansas state-added module 8: Outdoor Air Quality and Activity [24]. Additional questions were included to ascertain relevant demographic (age, gender) and health information (respiratory illness in the home). The survey questions as they were phrased for the phone-based survey are listed in Spanish in Additional file 1.

### Statistical analysis

Data were recorded, shared, and analyzed using SPSS Version 23.0 (released 2015, IBM Corp., Armonk, NY) and Graphpad Prism Version 6.0 g (released 2015, Graphpad Software Inc., La Jolla, CA). Sample errors were calculated using the formula (1.96 * √([pq/n])) * 100% and reported when appropriate. Unpaired t-tests were performed to determine statistical significance of air quality index awareness between responders with and without respiratory disease in the home, at a *p*-value of 0.05.

## Results

Demographic information recorded from the 803 participating respondents is summarized in Table 1 and indicates that age and gender of respondents were comparable between the two surveyed areas. The response rate of the survey was 21% which compares favorably to the average response rate of phone-based surveys administered in the United States [25]. Table 2 illustrates the occupations held by respondents, the top three being housewives, workers in the private sector, and students. The demographic information recorded by respondents in terms of gender and age distribution mirrors that of publically available MCMA census data.

**Table 1:** Survey Participant Demographics

MCMA Regions		Gender	
		Male	Female
Mexico City	18 to 25 years old	58	30
		13.6%	7.0%
	26 to 35 years old	30	34
		7.0%	8.0%
	36 to 45 years old	38	31
		8.9%	7.3%
	46 to 55 years old	20	38
		4.7%	8.9%
	56 years or more	51	97
		11.9%	22.7%
	Total	197	230
		46.1%	53.9%
State of Mexico	18 to 25 years old	55	31
		14.6%	8.2%
	26 to 35 years old	33	28
		8.8%	7.4%
	36 to 45 years old	36	33
		9.6%	8.8%
	46 to 55 years old	37	36
		9.8%	9.6%
	56 years or more	43	44
		11.4%	11.7%
	Total	204	172
		54.3%	45.7%

*Note.* Percentages reflect proportions of total respondents per age and gender group

*Abbreviations: MCMA* Mexico City Metropolitan Area

**Table 2:** Survey Participants Occupations

Occupation	Gender		Total
	Male	Female	
Housewife	7	222	229 (28.5%)
Private Sector	123	45	168 (20.9%)
Student	53	31	84 (10.5%)
Merchant	43	13	56 (7.0%)
Government Worker	30	25	55 (6.8%)
Self-employed	40	12	52 (6.5%)
Retired or pensioned	33	12	45 (5.6%)
Unemployed	23	5	28 (3.5%)
Independent Professional	15	8	23 (2.9%)
Entrepreneur	5	1	6 (0.7%)
Farmer or laborer	6	0	6 (0.7%)
Other	23	28	51 (6.4%)
Total	401	402	803

*Note.* Percentages reflect proportions of total respondents per occupation group

**Fig. 2:**
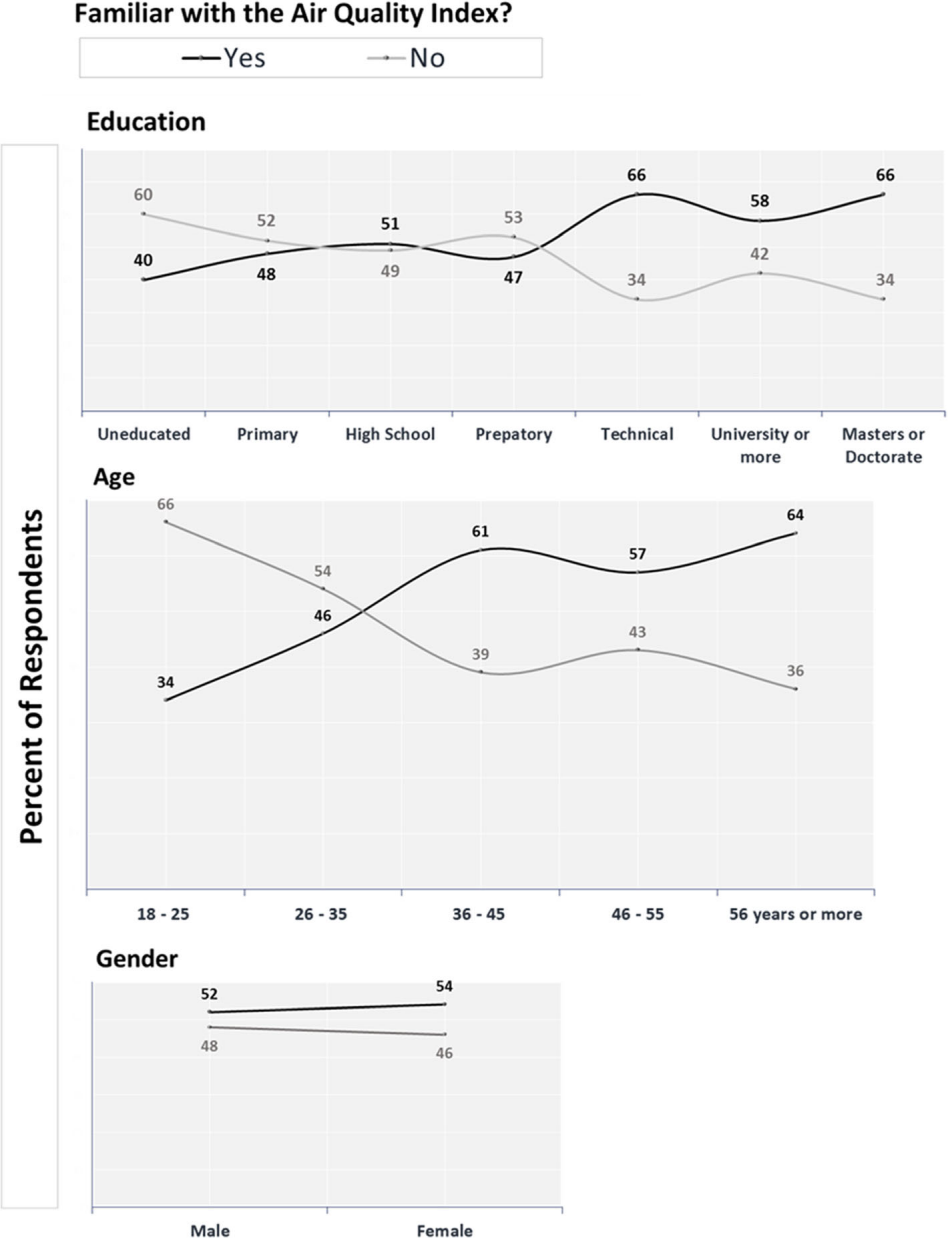
Respondents Familiar and Unfamiliar with Air Quality Index by Demographic. Figure shows a breakdown of respondents’ familiarity with the air quality index by demographics, including education, age, and gender. Dark lines reflect percentages of those in each category familiar with the air quality index; light lines show percentages of those unfamiliar with the index

Beyond participant demographic information, it was found that 15.5% of respondents either had a respiratory illness or a family member with a respiratory illness living in their household. This was determined through a question asking if a responder, or anyone in their home, had been diagnosed with a respiratory illness such as asthma. There was no difference in the proportion of respondents with respiratory illness in the home in Mexico City and the State of Mexico. Furthermore, Table 3 presents the air quality index awareness among both the entire metropolitan area (53.2%) and broken down by district. Note the increased awareness of IMECA among individuals living in Mexico City (61.4%) compared to the State of Mexico (43.9%). While results reveal no difference between air quality index awareness among males and females, there was a direct relationship with both age and education level (see Fig. 2).

**Table 3:** Familiarity with IMECA by Region and Respiratory Illness Status

MCMA Regions			Familiar with Air Quality Index?		Total
			Yes	No	
Mexico City	Respiratory Illness	Yes	47	20	67
			11.0%	4.7%	15.7%
		No	215	145	360
			50.4%	34.0%	84.3%
	Total		262	165	427
			61.4%	38.6%	
State of Mexico	Respiratory Illness	Yes	34	23	57
			9.0%	6.1%	15.2%
		No	131	188	319
			34.8%	50.0%	84.8%
	Total		165	211	376
			43.9%	56.1%	
Total	Respiratory Illness	Yes	81	43	124
			10.1%	5.4%	15.4%
		No	346	333	679
			43.1%	41.5%	84.6%
	Total		427	376	803
			53.2%	46.8%	

*Note.* Percentages reflect proportions of respondents by illness status and familiarity with the air quality index groups in the specified region

*Abbreviations: IMECA* The Índice Metropolitano de la Calidad del Aire, Mexico City’s air quality index, *MCMA* Mexico City Metropolitan Area

**Fig. 3:**
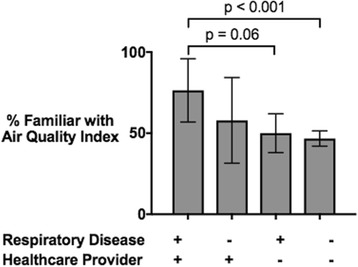
Familiarity with Air Quality Index by Respiratory Disease Status and Healthcare Provider Intervention. Graph shows percent of total respondents in each category that were familiar with the air quality index. Error bars show standard error. Four groups are compared, separated by those with (+) and without (−) a respiratory disease themselves or in a member of their household, and by those whose healthcare providers have (+) or have not (−) provided them with information about the risks of air quality. The brackets indicate significant differences between groups, labeled with their corresponding *p*-values

Subsequent analyses focused on what factors might influence familiarity with the air quality index apart from age and education. Results indicate that respondents with a respiratory illness themselves or in the home, compared to those who did not, were 14% more likely to be aware of the index, a significant result based on a two-tailed t-test (*p* < 0.001). Similarly, if a healthcare provider had specifically discussed air quality or air quality reports with the survey participant, the individual was significantly more likely to have knowledge of the index. Respondents with a respiratory illness in the home were more likely to have had a healthcare provider discuss the air quality index with them. Figure 3 compares index awareness based on both respiratory disease status and having a healthcare provider offer information about air quality risks to the respondent. Using two-tailed, unpaired parametric t-tests, analysis shows a significant difference between one pair of these groups. Specifically, those who received air quality information from their doctors and also had a positive respiratory disease status differed significantly from responders negative for respiratory disease who never had a healthcare provider discuss air quality risks with them (*p*-value of < 0.0001).

Following the assessment of air quality index awareness, analyses considered whether those who knew of this resource modified their behavior to reduce exposure to air pollution. To gauge this application of IMECA knowledge among respondents, the number of days modified, defined as avoiding exercise or strenuous activity outdoors in response to poor air quality over a 12-month period, was assessed. Table 4 shows the median number of days modified by disease status and familiarity with the air quality index. Results show that an awareness of IMECA led to a mild increase in median days modified, while there was no difference in behavior attributable to the presence of respiratory disease. Table 5 displays days modified annually by either perceived air pollution, or in response to an air quality alert. Perceived air pollution behavior modification was further separated by air quality index awareness status. It was found that 23.8% of respondents modified their behavior based on perceived poor air quality and were familiar with the index, and only 11.2% of respondents modified their behavior and were unfamiliar with this tool. Finally, 26.2% of respondents modified their behavior in response to an air quality report at least once over the course of 1 year.

**Table 4:** Days of Modified Behavior in Response to Perceived Air Quality by Respiratory Disease Status and IMECA Awareness

	Respiratory Disease +	Respiratory Disease -
Familiar with Air Quality Index	4.5 days (*n* = 50)	4 days (*n* = 141)
Unfamiliar with Air Quality Index	3 days (*n* = 21)	3 days (*n* = 69)

*Note.* Disease status is separated by those with (+) and without (−) a respiratory disease themselves or in a member of their household. N refers to the number of respondents in each category. Days reflect median values of annual days where behavior was modified in response to perceived air quality

*Abbreviations: IMECA* The Índice Metropolitano de la Calidad del Aire, Mexico City’s air quality index

**Table 5:** Days of Modified Behavior in Response to Poor Air Quality and to Air Quality Index or Alerts

		Days Modified in Response to Self-Perceived Air Quality					Total
		*0* *days*	*1–2* *days*	*3–4* *days*	*5–6* *days*	*7+* *days*	
Familiar with Air Quality Index?	Yes	236	58	47	26	60	427
		29.4%	7.2%	5.9%	3.2%	7.5%	53.2%
	No	286	37	27	8	18	376
		35.6%	4.6%	3.4%	1.0%	2.2%	46.8%
Total		522	95	74	34	78	803
		65.0%	11.8%	9.2%	4.2%	9.7%	100.0%
		Days Modified in Response to Air Quality Index or Alerts					Total
		*0* *days*	*1–2* *days*	*3–4* *days*	*5–6* *days*	*7+* *days*	
		217	67	59	35	49	427
		27.0%	8.3%	7.3%	4.4%	6.1%	53.2%

*Note.* Days modified are the number of days where behavior was modified annually. All percentages are reported for the entire study population of 803 participants. Perceived air quality is defined as days modified based on the responder's own interpretation of poor air quality independent of air quality index awareness. Days modified in response to air quality index or alerts was assessed only for those familiar with the Air Quality Index

## Discussion

A major purpose of air quality indices is to inform the population of the possible adverse health effects associated with current air quality conditions. Such reports also inform susceptible populations of behavior modifications necessary to reduce exposure to air pollution [26]. To date, few studies have been initiated to assess the public’s awareness of air quality indices [27, 28]. Even fewer studies have gone on to investigate if knowledge of these reports leads to more effective behavior modification in response to air pollution [26].

The results of this survey indicate a high general awareness of air quality reports in Mexico City, with 53% of all respondents in the MCMA reporting awareness of IMECA. Within the more suburban area of the State of Mexico, 44% of people surveyed had a knowledge of the index. Interestingly, this number is very close to the percentage of respondents (43%) who were index-aware in the 2005 BRFSS study in the state of Kansas, a rural area within the United States [22]. In contrast, of the 427 respondents living within Mexico City, 61% were familiar with their local air quality index. These data suggest that living within more urban areas may correlate with increased index awareness.

In addition to regional influences, other demographic characteristics and situational factors impacted air quality index knowledge. Results from the present study showed both higher education level and increased age were associated with a greater awareness of IMECA (see Fig. 2). Such data could be useful when considering campaigns to increase air quality report awareness by helping to narrow down which demographic groups to target with educational efforts.

A key part of the present survey analysis compared both the impact of household respiratory disease status and having a healthcare professional provide air quality information on an individual’s familiarity with the air quality index. While the presence of respiratory disease was significantly associated with knowledge of the air quality index, having a healthcare provider discuss air quality with the respondent was an even greater predictor of index awareness. Specifically, a comparison of the two groups with a positive respiratory disease status from Fig. 3 reveals that receiving information from a healthcare provider significantly increased knowledge of the air quality index for this susceptible population. This supports the conclusions from the 2005 Kansas BRFSS study, in which healthcare providers were shown to be an important factor in a respondent’s index knowledge [5]. Encouraging medical personnel to discuss air quality with their patients is thus one promising mechanism for improving the public’s awareness of air quality indices, particularly for subgroups most susceptible to pollution changes.

Results of the present study suggest behavior changes in response to air quality perceptions may not differ between responders with or without respiratory illness in the home. Given that respiratory illness-positive populations are more susceptible to the adverse health effects of air pollution, ideally they should display the largest number of days modified in response to air pollution. Yet between the four groups separated by respiratory disease status (+ or -) and air quality index awareness (familiar or unfamiliar), there were not meaningful differences in median days modified in response to perceived poor air quality (see Table 4). This signified that although respondents may have a knowledge of the index, there is not a measurable difference in exposure to poor air quality among the more susceptible population groups (i.e., those with respiratory illnesses). Given the potential differences in baseline activity levels between diseased and healthy populations, it is unclear at this point whether the lack of response by susceptible groups is a result of the current methodology of the AQI or a simply a lack of perceived benefits from behavior modification to reduce exposure to poor air quality. In the latter case, better explanations to those populations from their healthcare providers of the specific health benefits of additional activity modifications in response to air quality alerts would provide a simple solution, rather than a change the AQI construction itself. As such, future work will be needed to assess the true underlying cause of lack of response to AQI messages by those most at risk; such research may reveal room for improvement in air quality index construction and communication in order to ensure that the most susceptible individuals find utility in adhering to guidance from the index and are modifying behavior more frequently than those that are less susceptible.

The lack of a reported increase in behavior modification days in response to index values may be partially explained by a study conducted in Southern California, which found that people were most likely to modify their behavior and reduce outdoor activity during the first day of a poor air quality episode, while less likely to modify their behavior on subsequent days [29]. These results from California suggested that it was not a lack of reporting on air quality, but a lack of clear benefits gained that limited behavior modification to poor air quality. In addition to increased promotion of air quality index values, this result would indicate that greater attention may also need to be paid to whether real benefits are accruing for individuals adhering to guidance from air quality indices. Similarly, efforts to increase awareness of air quality indices may benefit from targeting susceptible individuals even within the traditional generalizations of children, the elderly, and people with underlying cardiovascular or respiratory diseases.

The existing literature suggests that an awareness of air quality reports does not guarantee that individuals will apply index recommendations. A survey of 240 parents with asthmatic children in a Salt Lake City, USA asthma clinic reported 88% of parents were aware of air quality reports; however, just half of the parents reduced children’s outdoor activity “sometimes” due to advisories, and only 7% of parents followed restrictions more than one third of the time [18]. In a study by Semenza et al. [19] from a survey in Portland, OR and Houston, TX, one third of respondents knew about the air quality index, but only 10–15% of participants modified their behavior based on index recommendations. Our results support this gap between knowledge and action, with 53% of respondents being aware of the air quality index, yet only 26% taking action to alter their behavior in response to these reports at least once annually.

When behavior change does occur, it is less often in response to air quality reports as it is to personal perception of poor air quality. In the MCMA, 35% of participants reported changes in behavior over one or more days annually in response to their own perception of poor air quality, compared to just 26% responding to air quality index reports. This influence of perception is in line with the Potter & Perveen [5] survey results, wherein participants modified their behavior predominantly due to perceived poor air quality and not air quality index reports. Often, time periods of perceived poor air quality did not overlap with times of measureable spikes in criteria air pollutants, suggesting behavior change based on perception is not as beneficial to individual health as changes made in response to data-driven reports [19, 20]. Considering the limited frequency of behavior modification in response to index recommendations suggests that the air quality index in its current format in the MCMA and abroad can be restructured to enhance usability and access to improve health benefits.

Future investigations on this topic would benefit from survey questions that directly assess how respondents obtain air quality information and what factors determine their perceptions of air quality. Specifically, questions could better gauge the means by which individuals learn about and access air quality indices, providing helpful information when considering ways to improve health risk communication to the public. A limitation to this study includes the generalizability to other areas of the world given that these data were collected in the MCMA. However, the data collected shows similar air quality index awareness to six U.S. states investigated in the 2005 BRFSS study [22] and is consistent with evidence from Zivin & Neidell [29] that behavioral modification to air pollution is most likely to occur on the first day of an episode with less avoidance behavior on subsequent days. This suggests that the informed risk of air quality does not result in lasting behavior modification and that it does not align with perceived risk from air pollution. In contrast, it was found that people living in Northern California and Nevada significantly modified their water consumption and purchased bottled water in response to warnings about tap water violations involving microorganisms and elements or chemicals [23]. The results of this study provide an example of a successful warning system in which the information provided resulted in a detectable behavior modification. The effectiveness of this program relative to an air quality index should be considered further, and may potentially be associated with differences in perceived risk and opportunity cost. Further research should focus on determining the ways individuals perceive and personally define poor air quality and how these perceptions influence behavior modification. Given such concerns are subject to temporal changes, it may be prudent to administer these surveys at different times of the year to see if seasonal variation alters responses and concerns about air quality.

## Conclusion

Air quality reports provide important information to the public regarding daily risks from air pollution. However, the existence of these reports alone is not enough to reduce the negative impacts of air pollution; this occurs only when individuals are both aware of air quality indices and choose to apply its guidance in their behavior decisions. Results of the present survey suggest healthcare providers can play an important role in promoting air quality index awareness, particularly among those with respiratory illnesses who are most susceptible to pollution changes. Beyond increasing the public’s awareness of air quality reports, indices themselves may benefit from improved construction that more strongly encourage individuals to rely on index information instead of personal perception of air quality. Future collaborations with social scientists should further investigate whether the lack of response to air quality index reports among susceptible groups is a direct result of index design itself, or whether it is impacted by other quantitative variables such as improved environmental health literacy. These points of intervention could be important tools for reducing the negative health effects of air pollution in the MCMA and other locales.

## Additional file

Additional file 1: Survey Questions (Spanish) (PDF 73 kb)

## Abbreviations

AQI: Air Quality Index (United States)
BRFSS: Behavior risk factor surveillance systems
IMECA: Índice Metropolitano de la Calidad del Aire (MCMA’s air quality index)
MCMA: Mexico City Metropolitan Area
