# Supplementary material for: Assessing air quality index awareness and use in Mexico City
Source: BMC Public Health. 2018 Apr 23;18:538. doi: 10.1186/s12889-018-5418-5 (PMC5913808; doi:10.1186/s12889-018-5418-5)
Supplement: Supplementary file 1 — Survey Questions (Spanish) (PDF 73 kb) [file 12889_2018_5418_MOESM1_ESM.pdf]

Additional File 1. Survey Questions (Spanish)

Buenos (as) días / tardes / noches / Mi nombre es_________________, le hablo de Parametría SA de CV, estamos haciendo una encuesta para conocer su opinión sobre temas ambientales. Quisiera hacerle unas preguntas. Su respuesta es confidencial y los datos serán utilizados con fines estadísticos. No le tomaremos más de 10 minutos. Gracias.

1. ¿Qué edad tiene? **(Suspender si es menor de 18 años)**

2. Género **(Anotar sin preguntar)**
   1) Masculino
   2) Femenino

3. ¿En qué delegación o municipio vive?

   Distrito Federal

   1) Álvaro Obregón
   2) Azcapotzalco
   3) Benito Juárez
   4) Coyoacán
   5) Cuajimalpa
   6) Cuauhtémoc
   7) Gustavo A. Madero
   8) Iztacalco
   9) Iztapalapa
   10) Magdalena Contreras
   11) Miguel Hidalgo
   12) Milpa Alta
   13) Tláhuac
   14) Tlalpan
   15) Venustiano Carranza
   16) Xochimilco

   Estado de México

   17) Acolman
   18) Atenco
   19) Atizapán de Zaragoza
   20) Chalco
   21) Chicoloapan
   22) Chimalhuacán
   23) Coacalco
   24) Cuautitlán
   25) Cuautitlán Izcalli
   26) Ecatepec
   27) Huixquilucan
   28) Ixtapaluca
   29) Jaltenco
   30) La Paz
   31) Melchor Ocampo
   32) Naucalpan
   33) Nezahualcóyotl
   34) Nextlalpan
   35) Tecámac
   36) Teoloyucán
   37) Tepotzotlán
   38) Texcoco
   39) Tlalnepantla
   40) Tonanitla
   41) Tultepec
   42) Tultitlán
   43) Valle de Chalco

4. ¿Hasta qué año estudió usted?
   1) Nada / Sin estudios
   2) Primaria
   3) Secundaria
   4) Preparatoria
   5) Normal / Carrera Técnica o comercial
   6) Universidad
   7) Maestría o doctorado

5. ¿Cuál es su principal ocupación?
   1) Trabajador en el gobierno
   2) Trabajador en sector privado
   3) Profesionista independiente
   4) Trabajador por cuenta propia
   5) Estudiante
   6) Comerciante
   7) Empresario
   8) Ama de casa
   9) Desempleado
   10) Jubilado o pensionado
   11) Campesino o jornalero
   12) Otro

6. ¿Qué medio de transportes utiliza para ir a su trabajo o escuela o realizar sus actividades cotidianas? **(Enc: registre todas las opciones que mencione)**

(…)	Si mencionó	No mencionó
a) Auto propio	1	2
b) Auto de la empresa	1	2
c) Auto del gobierno	1	2
d) Taxi	1	2
e) Microbús	1	2
f) Autobús	1	2
g) Metro	1	2
h) Combi	1	2
i) Metrobús	1	2
j) Tren ligero	1	2
k) Tren suburbano	1	2
l) Trolebús	1	2
m) Motocicleta	1	2
n) Bicicleta	1	2
o) Caminando	1	2
p) Ninguno	1	2

7. ¿Alguien en su hogar ha sido diagnosticado con asma o algún otro tipo de enfermedad respiratoria?
   1) Sí
   2) No

8. El gobierno adquiere de manera sistemática información sobre la calidad del aire, esto puede ayudar a informar al público acerca de la contaminación del medio ambiente. ¿Alguna vez ha escuchado o leído sobre el índice de calidad del aire donde vive?
   1) Sí **PASE A 9**
   2) No **PASE A 10**

9. Pensando en los últimos doce meses: ¿Cuántas veces redujo o cambió su nivel de actividad al aire libre debido al índice de calidad del aire o a alertas sobre la calidad del aire? Por ejemplo: evitar ejercicio o evitar una actividad agotadora al aire libre

10. Pensando en los últimos doce meses: ¿Cuantas veces usted o alguien de su familia redujo o cambió su nivel de actividad al aire libre debido a la mala calidad del aire o porque se sintió mal debido al aire que respiraba?

11. ¿Algún médico, enfermero u otro profesional de la salud le ha dicho a usted, u otra persona en su hogar, que redujeran su nivel de actividad física al aire libre cuando la calidad del aire fuera mala?
    1) Sí
    2) No

12. Tomando en cuenta que cierta información sobre la calidad del aire podría modificar su comportamiento e influenciar sus actividades cotidianas: ¿Que tipo de información le sería más útil? **(Encuestador: Leer opciones 1 y 2)**
    1. Pronósticos de la calidad del aire con un día de anticipación
    2. Condiciones de la calidad del aire en tiempo real
    3. Los dos **(Espontánea)**
    4. Ninguno **(Espontánea)**

13. En una escala del 1 al 10, donde 1 es que no está nada preocupado y 10 que está sumamente preocupado, ¿Cómo calificaría su preocupación por la contaminación en el Área Metropolitana de la Ciudad de México?

1	2	3	4	5	6	7	8	9	10

14. ¿Durante que periodo del año le preocupa más la contaminación?
    1. Durante los meses de Invierno (Diciembre-Febrero)
    2. Finales de Primavera (Abril – Junio)
    3. Meses de Verano (Junio – Agosto)
    4. Meses de Otoño (Septiembre – Noviembre)
    5. Me preocupa de manera igual durante todo el año
    6. No me preocupa la contaminación

15. ¿Durante que momento del día le preocupa más la contaminación?
    1. En la mañana, durante las horas pico
    2. A fin de mediodía, hasta las horas pico
    3. En la tarde
    4. Me preocupa de manera igual durante todo el día
    5. No me preocupa a ningún momento del día

16. ¿Le preocupa mas la contaminación durante los días entre semana (Lunes a Viernes) o en fin de semana (Sábado y Domingo)?
    1) Lunes a viernes
    2) Fin de semana
    3) Ambos **(Espontánea)**

17. ¿Qué aspecto de la contaminación le preocupa más?
(Encuestador: Leer opciones 1 a 4)

1. Visibilidad reducida
2. La salud personal
3. La salud de mi familia
4. Reducir las actividades al aire libre
5. Otro ____
6. Ningún aspecto de la contaminación le preocupa
